# Robust Biometrics Based Authentication and Key Agreement Scheme for Multi-Server Environments Using Smart Cards

**DOI:** 10.1371/journal.pone.0126323

**Published:** 2015-05-15

**Authors:** Yanrong Lu, Lixiang Li, Xing Yang, Yixian Yang

**Affiliations:** 1 Information Security Center, State Key Laboratory of Networking and Switching Technology, Beijing University of Posts and Telecommunications, Beijing 100876, China; 2 National Engineering Laboratory for Disaster Backup and Recovery, Beijing University of Posts and Telecommunications, Beijing 100876, China; Beihang University, CHINA

## Abstract

Biometrics authenticated schemes using smart cards have attracted much attention in multi-server environments. Several schemes of this type where proposed in the past. However, many of them were found to have some design flaws. This paper concentrates on the security weaknesses of the three-factor authentication scheme by Mishra et al. After careful analysis, we find their scheme does not really resist replay attack while failing to provide an efficient password change phase. We further propose an improvement of Mishra et al.’s scheme with the purpose of preventing the security threats of their scheme. We demonstrate the proposed scheme is given to strong authentication against several attacks including attacks shown in the original scheme. In addition, we compare the performance and functionality with other multi-server authenticated key schemes.

## Introduction

With the swift development of wireless communications and network technologies, more and more people use wireless handheld devices (e.g.PDA, notebook and mobile phone, etc) to enjoy mobile services almost anytime and anywhere. However, open nature of networks demands for security concern of paid and protected resources available over the network [1–5]. Authentication mechanism becomes an essential need before a remote user can access the services. Since then Lamport [6] proposed the first authentication scheme, a number of authentication schemes have been put forward for different applications [7–13].

However, most of the existing password authentication schemes are based on a single-server environment which are unfit for the multi-server environments. Recently, a large number of smart cards based remote user authentication schemes for multi-server environments have been proposed. In addition, compared with other authentication schemes, schemes that only use random numbers and a hash function were getting much more attention because of their low computation costs. In 2008, Tsai [14] proposed an efficient multi-server authentication scheme using the random number and one-way hash function. After that, numerous authenticated key agreement schemes were presented for multi-server environments one after another [15–17]. In 2012, Li et al. [18] proposed a novel authenticated key exchange scheme for multi-server environments. Unfortunately, Xue et al.[19] showed that Li et al.’s scheme did not resist some types of known attacks, such as vulnerability to verifier stolen, off-line password guess, replay, denial of service and forgery attacks. Then, Xue et al. proposed an improved scheme to remedy the weaknesses of Li et al.’s scheme. Nevertheless, Lu et al.[20] observed that Xue et al.’s scheme was not only really insecure against masquerade and insider attacks but also was vulnerable to off-line password guessing attack. To improve the shortcomings of Xue et al.’s scheme, Lu et al. proposed a slight modified authentication scheme for multi-server environments.

All above mentioned authentication schemes are based on password and smart cards. Note that the password cannot be considered as a unique identity identifier and it’s needed to be remembered. Moreover, possibility of password guessing attack is also a concern. Compared with cryptographic keys and passwords, biometric keys (e.g.fingerprint, face, iris, hand geometry and palm-print, etc.) have many advantages [21], for example, they are difficult to lose or forget; they are difficult to copy or share; they are difficult to forge or distribute biometrics; they are difficult to guess; they are more difficult to break biometric keys. Recently, Chuang et al.[22] presented an efficient biometrics based authentication scheme using smart cards for multi-server environments, which was previously considered to be have more security properties. However, Mishra et al. [23] showed that Chuang et al.’s scheme was vulnerable to stolen smart card attack, server spoofing attack and impersonation attack. In addition, they proposed an improved biometrics-based multi-server authenticated key agreement scheme using smart cards and they claimed that their scheme satisfied all desirable security requirements. Unfortunately, this paper will demonstrate that the scheme cannot really resist replay attack and cannot provide an efficient password change phase.

In this paper, we concentrate on the security weaknesses of the three-factor authentication scheme by Mishra et al. After carefully analysis, we find their scheme does not really resist replay attack while fails to provide an efficient password change phase. We further propose an improvement of Mishra et al.’s scheme with the purpose of preventing the security threats of their scheme. We demonstrate the proposed scheme is given to strong authentication against several attacks including attacks showed in the original scheme. In addition, we compare the performance and functionality with other related schemes.

The rest of paper is organized as follows: In Section 2 and Section 3, we review and analyze the Mishra et al.’s scheme. In Section 4, we propose an enhancement authentication scheme for multi-sever environments. In Section 5, we present a security analysis of our scheme. Section 6 shows security and performance analyses by comparing our scheme with previous schemes. We conclude in Section 7.

## Review of Mishra et al.’s scheme

There are three phases relating to Mishra et al.’s scheme which consists of the registration, login and authentication and password updating. Table 1 lists the notations used in this paper.

**Table 1:** Notations.

*U*_*i*_, *S*_*j*_	User, server
*RC*	The registration center
*ID*_*i*_, *SID*_*j*_	Identity of *U*_*i*_, *S*_*j*_
*PW*_*i*_, *BIO*_*i*_	Password and biometrics of *U*_*i*_
*x*, *y*	Master secret key of *U*_*i*_ and *RC*
*PSK*	Secure key shared by *RC* and *S*_*j*_
*h*(⋅)	Hash function
*H*(⋅)	Biohash function
⊕, ∣∣	Exclusive-or operation and concatenation operation

### Registration

Suppose *RC* is the trusted third party responsible for registration of *U*_*i*_ and *S*_*j*_.

#### Server registration

1. *S*_*j*_ sends the registration request to *RC*;
2. After receiving the request, *RC* sends the key *PSK* to *S*_*j*_ through a secure channel;
3. Upon receiving the secret key *PSK*, *S*_*j*_ stores it with aim to authorize a legitimate user.

#### User registration

1. *U*_*i*_ selects his identity *ID*_*i*_, password *PW*_*i*_ and keys his biometrics *BIO*_*i*_. Then, *U*_*i*_ generates a random number *N*_*i*_, computes *W*_1_ = *h*(*PW*_*i*_∣∣*N*_*i*_), *W*_2_ = *h*(*ID*_*i*_⊕*N*_*i*_) and sends the registration message {*ID*_*i*_, *W*_1_, *W*_2_} to *RC* via a secure channel.
2. *RC* computes *A*_*i*_ = *h*(*ID*_*i*_∣∣*x*∣∣*T*_*r*_), *B*_*i*_ = *h*(*A*_*i*_), *X*_*i*_ = *W*_*i*_⊕*B*_*i*_, *Y*_*i*_ = *h*(*PSK*)⊕*W*_2_ and *Z*_*i*_ = *PSK*⊕*A*_*i*_, where *T*_*r*_ is the registration time. Then, *RC* issues the smart card *SC*_*i*_ to *U*_*i*_ which contains {*X*_*i*_, *Y*_*i*_, *Z*_*i*_, *h*(⋅)} over a secure channel.
3. Upon receiving *SC*_*i*_, *U*_*i*_ enters his personal biometric *BIO*_*i*_ at the sensor and computes *N* = *N*_*i*_⊕*H*(*BIO*_*i*_), *V* = *h*(*ID*_*i*_∣∣*N*_*i*_∣∣*PW*_*i*_). Finally, *U*_*i*_ stores {*X*_*i*_, *Y*_*i*_, *Z*_*i*_, *N*, *V*, *h*(⋅)} into *SC*_*i*_.

### Login and authentication

1. *U*_*i*_ inserts *SC*_*i*_ into the terminal and inputs his identity *ID*_*i*_, password *PW*_*i*_ and imprints his biometrics *BIO*_*i*_ at the sensor.
2. *SC*_*i*_ computes *N*_*i*_ = *N*⊕*h*(*BIO*_*i*_) and checks $h(ID_{i}||N_{i}||PW_{i})\overset{?}{=}V$. If it holds, *SC*_*i*_ continues to compute *W*_1_ = *h*(*PW*_*i*_∣∣*N*_*i*_), *W*_2_ = *h*(*ID*_*i*_⊕*N*_*i*_), *B*_*i*_ = *X*_*i*_⊕*W*_*i*_ and *h*(*PSK*) = *Y*_*i*_⊕*W*_2_. Then, *SC*_*i*_ generates a random number *n*_1_ and computes *M*_1_ = *h*(*PSK*)⊕*n*_1_, *M*_2_ = *ID*_*i*_⊕*h*(*n*_1_∣∣*B*_*i*_) and *M*_3_ = *h*(*ID*_*i*_∣∣*n*_1_∣∣*B*_*i*_). Finally, *U*_*i*_ sends {*Z*_*i*_, *M*_1_, *M*_2_, *M*_3_} to *S*_*j*_.
3. When receiving the message from *SC*_*i*_, *S*_*j*_ immediately computes *A*_*i*_ = *Z*_*i*_⊕*PSK*, *n*_1_ = *M*_1_⊕*h*(*PSK*), *ID*_*i*_ = *M*_2_⊕*h*(*n*_1_∣∣*h*(*A*_*i*_)) and checks whether $h(n_{1}||B_{i}||ID_{i})\overset{?}{=}M_{3}$. If it is equal, *S*_*j*_ generates a random number *n*_2_ and computes *SK*_*ji*_ = *h*(*ID*_*i*_∣∣*SID*_*j*_∣∣*B*_*i*_∣∣*n*_1_∣∣*n*_2_), *M*_4_ = *n*_2_⊕*h*(*ID*_*i*_∣∣*n*_1_), *M*_5_ = *h*(*SK*_*ji*_∣∣*n*_1_∣∣*n*_2_). Then, *S*_*j*_ sends {*SID*_*j*_, *M*_4_, *M*_5_} to *SC*_*i*_.
4. *SC*_*i*_ first computes *n*_2_ = *M*_4_⊕*h*(*ID*_*i*_∣∣*n*_1_), *SK*_*ij*_ = *h*(*ID*_*i*_∣∣*SID*_*j*_∣∣*B*_*i*_∣∣*n*_1_∣∣*n*_2_) and then checks whether *h*(*SK*_*ij*_∣∣*n*_1_∣∣*n*_2_) is consistent with *M*_5_. If it is true, *SC*_*i*_ computes *M*_6_ = *h*(*SK*_*ij*_∣∣*n*_1_∣∣*n*_2_) and delivers it to *S*_*j*_.
5. *S*_*j*_ verifies the verification condition $M_{6}\overset{?}{=}h(SK_{ji}||n_{1}||n_{2})$. If this verification holds, *S*_*j*_ can now use the keys *SK*_*ji*_ to communicate with *U*_*i*_ securely.

### Password updating

*U*_*i*_ inputs his *ID*_*i*_, *PW*_*i*_ and imprints his biometrics *BIO*_*i*_ at the sensor. *SC*_*i*_ computes *N*_*i*_ = *N*⊕*h*(*BIO*_*i*_) and checks $h(ID_{i}||N_{i}||PW_{i})\overset{?}{=}V$. If *SC*_*i*_ determines that they are equal, then *U*_*i*_ can key the new password $PW_{i}^{new}$. Subsequently, *SC*_*i*_ computes $W_{1}^{new}=h(PW_{i}^{new}||N_{i}),X_{i}^{new}=X_{i}⊕W_{1}⊕W_{1}^{new},V_{i}^{new}=h(ID_{i}||N_{i}||PW_{i}^{new})$ and replaces *X*_*i*_ and *V*_*i*_ with $X_{i}^{new}$ and $V_{i}^{new}$, respectively.

## Security analysis of Mishra et al.’s scheme

This section presents a cryptanalysis of a recently scheme proposed by Mishra et al. We show their scheme does not satisfy the key security attribute such as vulnerability to replay attack and incorrect password change phase. We assume that a malicious adversary 𝓐 has totally supervised the communication channel in login and session key establishment phases. In other words, 𝓐 has the capacity to intercept, insert, delete, refresh or update any information delivered between *U*_*i*_ and *S*_*j*_ [6].

### Not withstanding the replay attack

Suppose an adversary 𝓐 has intercepted a past login message {*Z*_*i*_, *M*_1_, *M*_2_, *M*_3_}. He is able to launch a replay attack and login to the server by resending the eavesdropped message {*Z*_*i*_, *M*_1_, *M*_2_, *M*_3_} to *S*_*j*_. In other words, the adversary without running the “Login phase”, sends the eavesdropped message {*Z*_*i*_, *M*_1_, *M*_2_, *M*_3_} to *S*_*j*_. In the “Login and authentication”, upon receiving the message {*Z*_*i*_, *M*_1_, *M*_2_, *M*_3_}, *S*_*j*_ computes *A*_*i*_ = *Z*_*i*_⊕*PSK*, *n*_1_ = *M*_1_⊕*h*(*PSK*), *ID*_*i*_ = *M*_2_⊕*h*(*n*_1_∣∣*h*(*A*_*i*_)), $M_{3}^{\prime }=h(n_{1}||B_{i}||ID_{i})$ and checks whether $M_{3}^{\prime }$ is equal to the received *M*_3_ or not. Since *M*_3_ and $M_{3}^{\prime }$ are equal, *S*_*j*_ will authenticate 𝓐 and 𝓐 will be able to login to *S*_*j*_. Thus, 𝓐 can easily login to *S*_*j*_ by re-sending an old login message. Since *S*_*j*_ does not check the freshness of the received login message {*Z*_*i*_, *M*_1_, *M*_2_, *M*_3_} and authenticate *U*_*i*_ in (3) of the “Login and authentication”, *S*_*j*_ will not be able to discover replay attack.

### Incorrect password change phase

The user *U*_*i*_ inserts his smart card into a card reader and enters his identity *ID*_*i*_, password *PW*_*i*_ and imprints his personal biometric *BIO*_*i*_ at the sensor corresponding to his smart card. Then smart card computes *N*_*i*_ = *N*⊕*h*(*BIO*_*i*_), $V_{i}^{\prime }=h(ID_{i}||N_{i}||PW_{i})$ and compares $V_{i}^{\prime }$ with the stored value of *V* in its memory to verify the legitimacy of *U*_*i*_. Once the authenticity of cardholder is verified then *U*_*i*_ can instruct smart card to change his password. Afterwards, smart card asks the cardholder to resubmit a new password $PW_{i}^{new}$, then *X*_*i*_ = *B*_*i*_⊕*h*(*PW*_*i*_∣∣*N*_*i*_) and *V* = *h*(*ID*_*i*_∣∣*N*_*i*_∣∣*PW*_*i*_) stored in the smart card can be updated with $X_{i}^{new}=X_{i}⊕W_{1}⊕W_{1}^{new}$ and $V_{i}^{new}=h(ID_{i}||N_{i}||PW_{i}^{new})$, where $W_{1}^{new}=h(PW_{i}^{new}||N_{i})$. The $X_{i}^{new}$ value contains older password *PW*_*i*_ in *h*(*PW*_*i*_∣∣*N*_*i*_). Therefore, the modified $X_{i}^{new}$ is not correct.

## The proposed scheme

**Fig 1:**
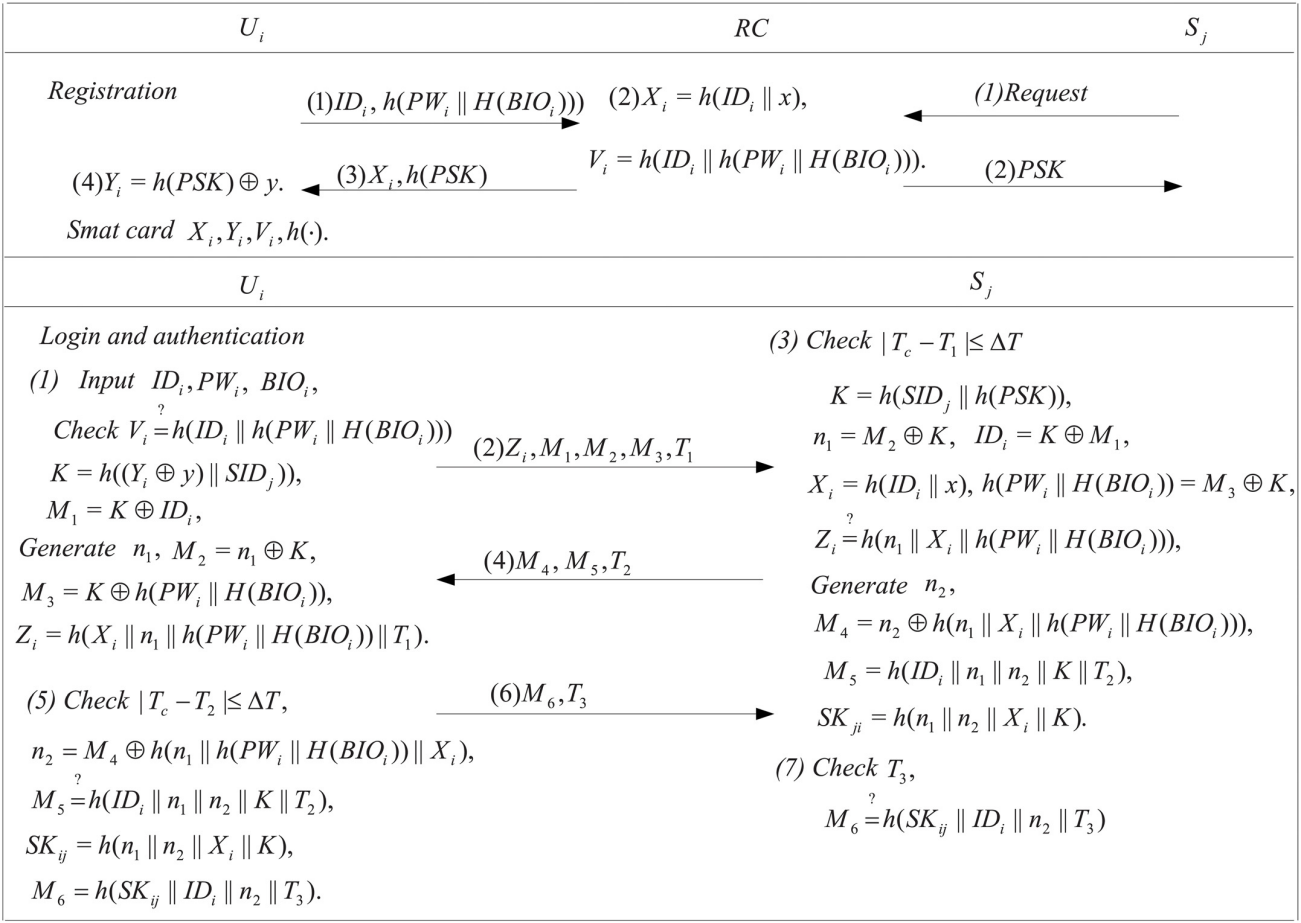
Registration and authentication phases.

In this section, we will present our robust biometrics based authentication scheme using smart cards for multi-sever environments. In our scheme, there are also three participants, the user *U*_*i*_, the server *S*_*j*_ and the registration center *RC*. *RC* chooses the secret key *PSK* and a secret number *x* and shares them with *S*_*j*_ via a secure channel. We will describe all the phases relating to our scheme in the subsections, i.e. registration, login and authentication, and password update, where registration and login and authentication phases are shown in Fig 1.

### Registration

1. *U*_*i*_ keys his biometrics *BIO*_*i*_, identity *ID*_*i*_ and password *PW*_*i*_. Then, *U*_*i*_ sends {*ID*_*i*_, *h*(*PW*_*i*_∣∣*H*(*BIO*_*i*_))} to *RC*.
2. Upon receiving the message from *U*_*i*_, *RC* computes *X*_*i*_ = *h*(*ID*_*i*_∣∣*x*), *V* = *h*(*ID*_*i*_∣∣*h*(*PW*_*i*_∣∣*H*(*BIO*_*i*_))). Then, *RC* stores {*X*_*i*_, *V*_*i*_, *h*(*PSK*)} into a smart card and submits them to *U*_*i*_.
3. *U*_*i*_ computes *Y*_*i*_ = *h*(*PSK*)⊕*y*, and replaces *h*(*PSK*) with *Y*_*i*_. Finally, the smart card stores the values of {*X*_*i*_, *Y*_*i*_, *V*_*i*_, *h*(⋅)}.

### Login and authentication

1. *U*_*i*_ inserts his smart card into device and enters his identity *ID*_*i*_, password *PW*_*i*_ and biometrics *BIO*_*i*_. Then, the smart card validates whether *V*_*i*_ = *h*(*ID*_*i*_∣∣*h*(*PW*_*i*_∣∣*H*(*BIO*_*i*_))) is equal to the stored *V*. If it holds, the smart card generates a random number *n*_1_ and computes *K* = *h*((*y*⊕*Y*_*i*_)∣∣*SID*_*j*_), *M*_1_ = *K*⊕*ID*_*i*_, *M*_2_ = *n*_1_⊕*K*, *M*_3_ = *h*(*PW*_*i*_∣∣*H*(*BIO*_*i*_))⊕*K*, *Z*_*i*_ = *h*(*B*_*i*_∣∣*n*_1_∣∣*h*(*PW*_*i*_∣∣*H*(*BIO*_*i*_))∣∣*T*_1_). Finally, *U*_*i*_ submits {*Z*_*i*_, *M*_1_, *M*_2_, *M*_3_, *T*_1_} to *S*_*j*_, where *T*_1_ is the current timestamp.
2. Upon receiving the message from *U*_*i*_, *S*_*j*_ first checks whether *T*_*c*_−*T*_1_ ≤ Δ*T* and then computes *K* = *h*(*SID*_*j*_∣∣*h*(*PSK*)) by using a secure pre-shared key *PSK*. Then, *S*_*j*_ retrieves *ID*_*i*_ = *M*_1_⊕*K*, *n*_1_ = *M*_2_⊕*K*, *h*(*PW*_*i*_∣∣*BIO*_*i*_) = *M*_3_⊕*K*. Now, *S*_*j*_ computes *X*_*i*_ = *h*(*ID*_*i*_∣∣*x*) and verifies whether $h(X_{i}||n_{1}||h(PW_{i}||H(BIO_{i})))\overset{?}{=}Z_{i}$. If it holds, *S*_*j*_ generates a random number *n*_2_ and computes *SK*_*ji*_ = *h*(*n*_1_∣∣*n*_2_∣∣*K*∣∣*X*_*i*_), *M*_4_ = *n*_2_⊕*h*(*n*_1_∣∣*h*(*PW*_*i*_∣∣*H*(*BIO*_*i*_))∣∣*X*_*i*_), *M*_5_ = *h*(*ID*_*i*_∣∣*n*_1_∣∣*n*_2_∣∣*K*∣∣*T*_2_). Then, *S*_*j*_ sends back authentication message {*M*_4_, *M*_5_, *T*_2_} to *U*_*i*_, where *T*_2_ is the current timestamp.
3. After checking the freshness of *T*_2_, *U*_*i*_ first computes *n*_2_ = *M*_4_⊕*h*(*n*_1_∣∣*h*(*PW*_*i*_∣∣*H*(*BIO*_*i*_))∣∣*X*_*i*_) and then verifies whether *h*(*ID*_*i*_∣∣*n*_1_∣∣*n*_2_∣∣*K*) is equal to the received *M*_5_. If they are equal, *U*_*i*_ computes the common session key *SK*_*ij*_ = *h*(*n*_1_∣∣*n*_2_∣∣*K*∣∣*X*_*i*_) and sends {*M*_6_ = *h*(*SK*_*ij*_∣∣*ID*_*i*_∣∣*n*_2_∣∣*T*_3_), *T*_3_} to *S*_*j*_, where *T*_3_ is the current timestamp.
4. *S*_*j*_ verifies the freshness *T*_3_ and the correctness of *M*_6_ by using *SK*_*ji*_. If they do not hold, *S*_*j*_ stops the execution; Otherwise, *S*_*j*_ confirms the common session key *SK*_*ji*_ with *U*_*i*_.

### Password updating

*U*_*i*_ first inputs his smart card into the device and provides his identity *ID*_*i*_, password *PW*_*i*_ and biometrics *BIO*_*i*_. Then, the smart card validates whether *V*_*i*_ = *h*(*ID*_*i*_∣∣*h*(*PW*_*i*_∣∣*H*(*BIO*_*i*_))) is equal to the stored *V*_*i*_. If they are not equal, the smart card refuses the request; Otherwise, *U*_*i*_ keys in the new password $PW_{i}^{new}$. Finally, the smart card computes $V_{i}^{new}=h(ID_{i}||h(PW_{i}^{new}||H(BIO_{i})))$ and replaces *V*_*i*_ by $V_{i}^{new}$.

## Security analysis of the proposed scheme

In this section, we first adopt Burrows-Abadi-Needham (BAN)Logic [24] to demonstrate the completeness of the proposed scheme. Then, we conduct discussion and a cryptanalysis of the proposed scheme through both the informal and formal analyses.

### Verifying the proposed scheme with BAN logic

BAN logic [24] is a set of rules for defining and analyzing information exchange schemes. It helps its users determine whether exchanged information is trustworthy, secured against eavesdropping, or both. It has been highly successful in analyzing the security of authentication schemes. First, we introduce some notations and logical postulates of BAN logic in Table 2.

1. BAN logical postulates
  - Message-meaning rule: $\frac{A|\equiv A\overset{K}{↔}B,A⊲<X{>}_{K}}{A|\equiv |B∼X}$: if *A* believes that the key *K* is shared by *A* and *B*, and sees *X* encrypted with *K*, then *A* believes that *B* once said *X*.
  - Fresh conjuncatenation rule: $\frac{A|\equiv \#(X)}{A|\equiv \#(X,Y)}$: if *A* believes freshness of *X*, then *A* believes freshness of the (*X*, *Y*).
  - Belief rule: $\frac{A|\equiv X,A|\equiv Y}{A|\equiv (X,Y)}$: if *A* believes *X* and *Y*, then *A* believes (*X*, *Y*).
  - Nonce-verification rule: $\frac{A|\equiv \#(X),A|\equiv B|∼X}{A|\equiv B|\equiv X}$: if *A* believes that *X* could have been uttered only recently and that *B* once said *X*, then *A* believes that *B* believes *X*.
  - Jurisdiction rule: $\frac{A|\equiv B\Rightarrow X,A|\equiv B|∼X}{A|\equiv X}$: if *A* believes that *B* has jurisdiction over *X* and *A* trusts *B* on the truth of *X*, then *A* believes *X*.
2. Establishment of security goals
$g_{1}.S_{j}|\equiv U_{i}|\equiv U_{i}\overset{SK_{ij}}{↔}S_{j}$
$g_{2}.S_{j}|\equiv U_{i}\overset{SK_{ij}}{↔}S_{j}$
$g_{3}.U_{i}|\equiv S_{j}|\equiv U_{i}\overset{SK_{ji}}{↔}S_{j}$
$g_{4}.U_{i}|\equiv U_{i}\overset{SK_{ji}}{↔}S_{j}$
3. Idealized scheme
$U_{i}:<n_{1},ID_{i},h(PW_{i}\|H(BIO_{i})){>}_{K},{\left(n_{1},X_{i},T_{1}\right)}_{h(PW_{i}\|H(BIO_{i}))},{\left(n_{2},U_{i}\overset{SK_{ij}}{↔}S_{j},T_{3}\right)}_{ID_{i}}$
*S*_*j*_: < *n*_1_, *X*_*i*_, *h*(*PW*_*i*_∣∣*H*(*BIO*_*i*_)) > *n*_2_, (*ID*_*i*_, *n*_1_, *n*_2_, *T*_2_)_*K*_
4. Initiative premises
*p*_1_. *U*_*i*_∣ ≡ #*n*_1_ *p*_2_. *U*_*i*_∣ ≡ *S*_*j*_ ⇒ #*n*_2_ *p*_3_. *S*_*j*_∣ ≡ #*n*_1_ *p*_4_. *S*_*j*_∣ ≡ #*n*_2_
$p_{5}.S_{j}|\equiv U_{i}\overset{K}{↔}S_{j}p_{6}.U_{i}|\equiv U_{i}\overset{K}{↔}S_{j}$
*p*_7_. *U*_*i*_∣ ≡ *ID*_*i*_ *p*_8_. *S*_*j*_∣ ≡ *U*_*i*_ ⇒ *h*(*PW*_*i*_∣∣*BIO*_*i*_)
*p*_9_. *S*_*j*_∣ ≡ *U*_*i*_ ⇒ *ID*_*i*_ *p*_10_. *U*_*i*_∣ ≡ *S*_*j*_ ⇒ *X*_*i*_
*p*_11_. $S_{j}|\equiv U_{i}\Rightarrow U_{i}\overset{SK_{ij}}{↔}S_{j}p_{12}$. $U_{i}|\equiv S_{j}\Rightarrow U_{i}\overset{SK_{ij}}{↔}S_{j}$
5. Scheme analysis
*a*_1_. By *p*_5_ and *S*_*j*_⊲ < *n*_1_, *ID*_*i*_, *h*(*PW*_*i*_∣∣*BIO*_*i*_) > *K*, we apply the message-meaning rule to derive: *S*_*j*_∣ ≡ *U*_*i*_∣ ∼ (*n*_1_, *ID*_*i*_, *h*(*PW*_*i*_∣∣*H*(*BIO*_*i*_)))
*a*_2_. By *a*_1_ and *p*_3_, we apply the fresh conjuncatenation rule and the nonce-verification rule to derive: *S*_*j*_∣ ≡ *U*_*i*_∣ ≡ (*n*_1_, *ID*_*i*_, *h*(*PW*_*i*_∣∣*H*(*BIO*_*i*_)))
*a*_3_. By *a*_2_, *p*_3_ and *p*_8_, we apply the belief rule and the jurisdiction rule to derive: *S*_*j*_∣ ≡ *ID*_*i*_
*a*_4_. By *a*_3_ and $S_{j}⊲{\left(n_{2},U_{i}\overset{SK_{ij},}{↔}S_{j},T_{3}\right)}_{ID_{i}}$, we apply the message-meaning rule to derive: $S_{j}|\equiv U_{i}|∼(n_{2},U_{i}\overset{SK_{ij}}{↔}S_{j},T_{3})$
*a*_5_. By *p*_4_ and *a*_4_, we apply the fresh conjuncatennation rule and the nonce-verification rule to derive: $S_{j}|\equiv U_{i}|\equiv (n_{2},U_{i}\overset{SK_{ij}}{↔}S_{j},T_{3})$
*g*_1_. By *a*_5_, we apply the belief rule to derive: $S_{j}|\equiv U_{i}|\equiv U_{i}\overset{SK_{ij}}{↔}S_{j}$
*g*_2_. By *g*_1_ and *p*_11_, we apply the jurisdiction rule to derive: $S_{j}|\equiv U_{i}\overset{SK_{ij}}{↔}S_{j}$
*a*_6_. By *p*_6_ and *U*_*i*_⊲(*ID*_*i*_, *n*_1_, *n*_2_, *T*_2_)_*K*_, we apply the message-meaning rule to derive: *U*_*i*_∣ ≡ *S*_*j*_∣ ∼ (*ID*_*i*_, *n*_1_, *n*_2_, *T*_2_)
*a*_7_. By *p*_2_ and *a*_9_, we apply the fresh conjuncatenation rule and the nonce-verification rule to derive: *U*_*i*_∣ ≡ *S*_*j*_∣ ≡ (*ID*_*i*_, *n*_1_, *n*_2_, *T*_2_)
*a*_8_. By *a*_7_, we apply the belief rule to derive: *U*_*i*_∣ ≡ *S*_*j*_∣ ≡ *n*_2_
*a*_9_. By *p*_2_ and *a*_8_, we apply the jurisdiction rule to derive: *U*_*i*_∣ ≡ *n*_2_
*a*_10_. By *a*_9_ and *U*_*i*_⊲ < *n*_1_, *X*_*i*_, *h*(*PW*_*i*_∣∣*BIO*_*i*_) > *n*_2_, we apply the message-meaning rule to derive: *U*_*i*_∣ ≡ *S*_*j*_∣ ∼ (*n*_1_, *X*_*i*_, *h*(*PW*_*i*_∣∣*BIO*_*i*_))
*a*_11_. By *a*_10_ and *p*_1_, we apply the fresh conjuncatennation rule and the nonce-verification rule to derive: *U*_*i*_∣ ≡ *S*_*j*_∣ ≡ (*n*_1_, *X*_*i*_, *h*(*PW*_*i*_∣∣*BIO*_*i*_))
*g*_3_. By *p*_1_, *p*_3_, *p*_4_, *p*_6_, *a*_11_ and *SK*_*ji*_ = *h*(*n*_1_∣∣*n*_2_∣∣*K*∣∣*X*_*i*_), we apply the fresh conjuncatennation rule and the nonce-verification rule to derive: $U_{i}|\equiv S_{j}|\equiv U_{i}\overset{SK_{ji}}{↔}S_{j}$
*g*_4_. By *g*_3_ and *p*_12_, we apply the jurisdiction rule to derive: $U_{i}|\equiv U_{i}\overset{SK_{ji}}{↔}S_{j}$

**Table 2:** BAN logic notations.

*A*∣ ≡ *X*	*A* believes a statement *X*
$A\overset{K}{↔}B$	Share a key *K* between *A* and *B*
#*X*	*X* is fresh
*A*⊲*X*	*A* sees *X*
*A* ⇒ *X*	*A* controls *X*
*A*∣ ∼ *X*	*A* said *X*
(*X*)_*K*_	The formula *X* is hashed by *K*
< *X*, *Y* > *K*	*X* and *Y* are encrypted with the key *K*
(*X*, *Y*)	The formula *X* or *Y* is one part of the formula (*X*, *Y*)

### Informal security analysis

This subsection verifies whether the proposed scheme is secure against various kinds of known attacks. We assume that a malicious adversary 𝓐 has totally supervised the communication channel in login and session key establishment phases. In other words, 𝓐 has the capacity to intercept, insert, delete, refresh or update any information delivered between *U*_*i*_ and *S*_*j*_ [6].

#### Anonymity

*U*_*i*_’s identity *ID*_*i*_ is well protected by the shared secret parameter *K* as a substitute for real ones, 𝓐 can not get users’ real identities. In addition, the unauthorized server cannot get *ID*_*i*_ without knowing *K* since *K* is protected by the secret key *PSK* only known by the authorized server and is not exposed in the open channel. Thus, our scheme provides user anonymity, which can prevent the leakage of private user identities to malicious attackers.

#### Mutual authentication

In order to authenticate *U*_*i*_, *S*_*j*_ has to verify validity of the evidence *Z*_*i*_ = *h*(*X*_*i*_∣∣*n*_1_∣∣*h*(*PW*_*i*_∣∣*H*(*BIO*_*i*_))). The evidence is computed with the common secret parameter *K* only known *U*_*i*_ and *S*_*j*_. In other words, (*n*_1_, *ID*_*i*_, *h*(*PW*_*i*_∣∣*H*(*BIO*_*i*_))) are derived from the valid login message {*Z*_*i*_, *M*_1_, *M*_2_, *M*_3_, *T*_1_} through *K*, no one can counterfeit the evidence. In addition, to compute *X*_*i*_, secret key *x* is needed but only known by *S*_*j*_. Moreover, checking *h*(*SK*_*ij*_∣∣*ID*_*i*_∣∣*n*_2_) to further assist *S*_*j*_ in authenticating *U*_*i*_ because the session key is only known by *U*_*i*_ and *S*_*j*_. To authenticate *S*_*j*_, *U*_*i*_ needs to verify whether $M_{5}\overset{?}{=}h(ID_{i}||n_{1}||n_{2}||K)$. Because *ID*_*i*_ and *K* are only known by *U*_*i*_ and *S*_*j*_, no one can forge a valid {*M*_4_, *M*_5_, *T*_2_} without them. Hence, mutual authentication between *U*_*i*_ and *S*_*j*_ is achieved.

#### Resist stolen smart card attack

Even if 𝓐 has gathered [25] the information {*X*_*i*_, *Y*_*i*_, *V*_*i*_, *h*(⋅)} stored in the smart card, 𝓐 cannot figure out the login request message {*Z*_*i*_, *M*_1_, *M*_2_, *M*_3_, *T*_1_} without the secret key *y*. Moreover, 𝓐 cannot get the identity *ID*_*i*_ and *PW*_*i*_ since they are protected by hash functions with the *U*_*i*_’s biometrics *BIO*_*i*_. Hence, 𝓐 still cannot succeed if he steals the smart card.

#### Session key agreement

We provide the session key *SK* = *h*(*n*_1_∣∣*n*_2_∣∣*K*∣∣*X*_*i*_) to protect the message communication between *U*_*i*_ and *S*_*j*_, where (*n*_1_, *n*_2_, *K*, *X*_*i*_) are known to anybody but *U*_*i*_ and *S*_*j*_. In addition, *SK* is different in each session, 𝓐 has obtained a known session key cannot be used to calculate the value of the next session key.

#### Resist replay attack

Assume 𝓐 has intercepted all the communication message {*Z*_*i*_, *M*_1_, *M*_2_, *M*_3_, *T*_1_, *M*_4_, *M*_5_, *T*_2_, *M*_6_, *T*_3_,} and tried to replay them to *U*_*i*_ or *S*_*j*_ to obtain authentication. However, it is impossible to come true since all the authenticated messages imply the timesstamp which is also exposed in public channel. If 𝓐 resends the transmitted messages, the receiver will immediately detect the attack through the authenticated message. Hence, our scheme can withstand replay attack.

#### Resist stolen verifier and insider attacks

In the registration phase, *RC* does not directly get the *U*_*i*_’s password *PW*_*i*_ and biometrics information *BIO*_*i*_. Hence, 𝓐 performs a stolen verifier attack or insider attack will be hard.

#### Resist off-line guessing attack

In our proposed scheme, trying to launch an off-line passsword guessing attack with the information stored in the smart card and the eavesdropped messages is trying to solve the input from the given hash value. Since the identity *ID*_*i*_ and the random number *N*_*i*_ are required with the purposed of knowing *PW*_*i*_, both the secrets are protected by the hash function and known by the user himself.

### Formal security analysis of the proposed scheme

This subsection presents the formal security analysis of our scheme and shows that it is secure. For this, we first define the following hash function [26].

**Definition 1**. A secure one-way hash function *h*:{0, 1}* → {0, 1}^*n*^, which takes an input as an arbitrary length binary string *x* ∈ {0,1}* and outputs a binary string *h*(*x*) ∈ {0,1}^*n*^ and satisfies the following requirements: *a*. Given *y* ∈ *Y*, it is computationally infeasible to find an *x* ∈ *X* such that *y* = *h*(*x*); *b*. Given *x* ∈ *X*, it is computationally infeasible to find another *x*′ ≠ *x* ∈ *X*, such that *h*(*x*′) = *h*(*x*); c. It is computationally infeasible to find a pair (*x*′, *x*) ∈ *X*′ × *X*, with *x*′ ≠ *x*, such that *h*(*x*′) = *h*(*x*).

**Theorem 1**. Under the assumption that the one-way hash function *h*(⋅) closely behaves like an oracle, then our scheme is provably secure against an attacker 𝓐 for protecting user’s personal information including identity *ID*_*i*_, password *PW*_*i*_ and biometrics *BIO*_*i*_, sever’s private key *x* and *PSK*.

**Proof**. The formal security proof of our scheme is similar to that as in [27–28]. Using the following oracle to construct 𝓐 who will have the ability to derive the user’s *ID*_*i*_, password *PW*_*i*_, biometrics *BIO*_*i*_, sever’s private key *x* and *PSK*.

Reveal: This random oracle will unconditionally output the input x from the given hash value *y* = *h*(*x*).

𝓐 runs the experimental algorithm showed in Table 3, $EXP_{HASH,𝓐}^{BAKASSCMSE}$ for our biometrics based authentication and key agreement scheme using smart cards for multi-server environments, say BAKASSCMSE.

**Table 3:** Algorithm 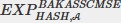.

1.	Eavesdrop login message {*Z*_*i*_, *M*_1_, *M*_2_, *M*_3_, *T*_1_}
2.	Call the Reveal oracle. Let $\left(X_{i}^{\prime },n_{1}^{\prime },p^{\prime })\leftarrow Reveal(Z_{i}\right)$
3.	Eavesdrop authentication message {*M*_4_, *M*_5_, *T*_2_}
4.	Call the Reveal oracle. Let $\left(ID_{i}^{\prime },n_{1}^{\prime \prime },n_{2}^{\prime },K^{\prime },T_{2})\leftarrow Reveal(M_{5}\right)$
5.	**if** $\left(n_{1}^{\prime }=n_{1}^{\prime \prime }\right)$ **then**
6.	Call the Reveal oracle. Let $\left(PW_{i}^{\prime },BIO_{i}^{\prime })\leftarrow Reveal(p^{\prime }\right)$
7.	Call the Reveal oracle. Let $\left(ID_{i}^{\prime },x^{\prime })\leftarrow Reveal(X_{i}^{\prime }\right)$
8.	Compute $K^{\prime \prime }=M_{2}⊕n_{1}^{\prime }$
9.	**if** (*K*′ = *K*′′) **then**
10.	Call the Reveal oracle. Let (*q*′, *SID*_*j*_) ← *Reveal*(*K*)
11.	Compute $n_{2}^{\prime \prime }=M_{4}⊕h(n_{1}^{\prime }||X_{i}||h^{\prime }(PW_{i}||BIO_{i}))$
12.	**if** $\left(n_{2}^{\prime }=n_{2}^{\prime \prime }\right)$ **then**
13.	Call the Reveal oracle. Let (*PSK*′) ← *Reveal*(*q*′)
14.	Accept $ID_{i}^{\prime },PW_{i}^{\prime },BIO_{i}^{\prime }$ as the correct *ID*_*i*_, *PW*_*i*_ and *BIO*_*i*_ of *U*_*i*_ *x*′ and *PSK*′ as the correct private key of *S*_*j*_
15.	**return** 1
16.	**else**
17.	**return** 0
18.	**end if**
19.	**else**
20.	**return** 0
21.	**end if**
22.	**else**
23.	**return** 0
24.	**end if**

Define the success probability for $EXP_{HASH,𝓐}^{BAKASSCMSE}$ is $Succ_{HASH,𝓐}^{BAKASSCMSE}=|Pr[EXP_{HASH,𝓐}^{BAKASSCMSE}=1]-1|$ and the advantage function for this experiment then becomes $Adv_{HASH,𝓐}^{BAKASSCMSE}(t,q_{R})=max_{𝓐}\backslash Succ_{HASH,𝓐}^{BAKASSCMSE}$, where the maximum is taken over all 𝓐 with execution time *t* and the number of queries *q*_*R*_ made to the Reveal oracle. Consider the experiment showed in Table 3 for 𝓐. If 𝓐 has the ability to solve the hash function problem provided in Definition 1, then he can directly derive *U*_*i*_’s identity *ID*_*i*_, password *PW*_*i*_, biometrics *BIO*_*i*_, and *S*_*j*_’s private key *x* and *PSK*. In this case, 𝓐 will discover the complete connections between *U*_*i*_ and *S*_*j*_. However, it is a computationally infeasible problem to invert the input from a given hash value, i.e., $Adv_{HASH,𝓐}^{BAKASSCMSE}(t)\leq \epsilon$, ∀*ϵ* > 0. Hence, we have $Adv_{HASH,𝓐}^{BAKASSCMSE}(t,q_{R})\leq \epsilon$, since $Adv_{HASH,𝓐}^{BAKASSCMSE}(t,q_{R})$ depends on $Adv_{HASH,𝓐}^{BAKASSCMSE}(t)$. As a result, there is no way for 𝓐 to discover the complete connections between *U*_*i*_ and *S*_*j*_ and our scheme is provably secure against an adversary for deriving (*ID*_*i*_, *PW*_*i*_, *BIO*_*i*_, *x*, *PSK*).

## Performance and functionality analysis

In this section, we compare our scheme with other existing multi-server authenticated schemes ([18–20], [22–23]) regarding security and performance. Table 4 lists the functionality comparisons of our proposed scheme with other related schemes. It can be seen that the proposed scheme achieves all security and functionality requirements and is more secure than other related schemes.

**Table 4:** Functionality comparison.

	Ours	Mishra et al. [23]	Chuang et al. [22]	Lu et al. [20]	Xue et al. [19]	Li et al. [18]
Provide mutual authentication	Yes	Yes	No	Yes	Yes	Yes
User anonymity	Yes	Yes	Yes	Yes	Yes	Yes
Resist insider attack	Yes	Yes	Yes	Yes	No	Yes
Resist off-line guessing attack	Yes	Yes	Yes	Yes	No	No
Resist stolen smart card attack	Yes	Yes	No	-	Yes	Yes
Resist replay attack	Yes	No	No	No	No	No
Resist verifier attack	Yes	Yes	Yes	-	No	Yes
Session key agreement	Yes	Yes	Yes	Yes	Yes	Yes
Efficient password change phase	Yes	No	No	Yes	No	No

**Fig 2:**
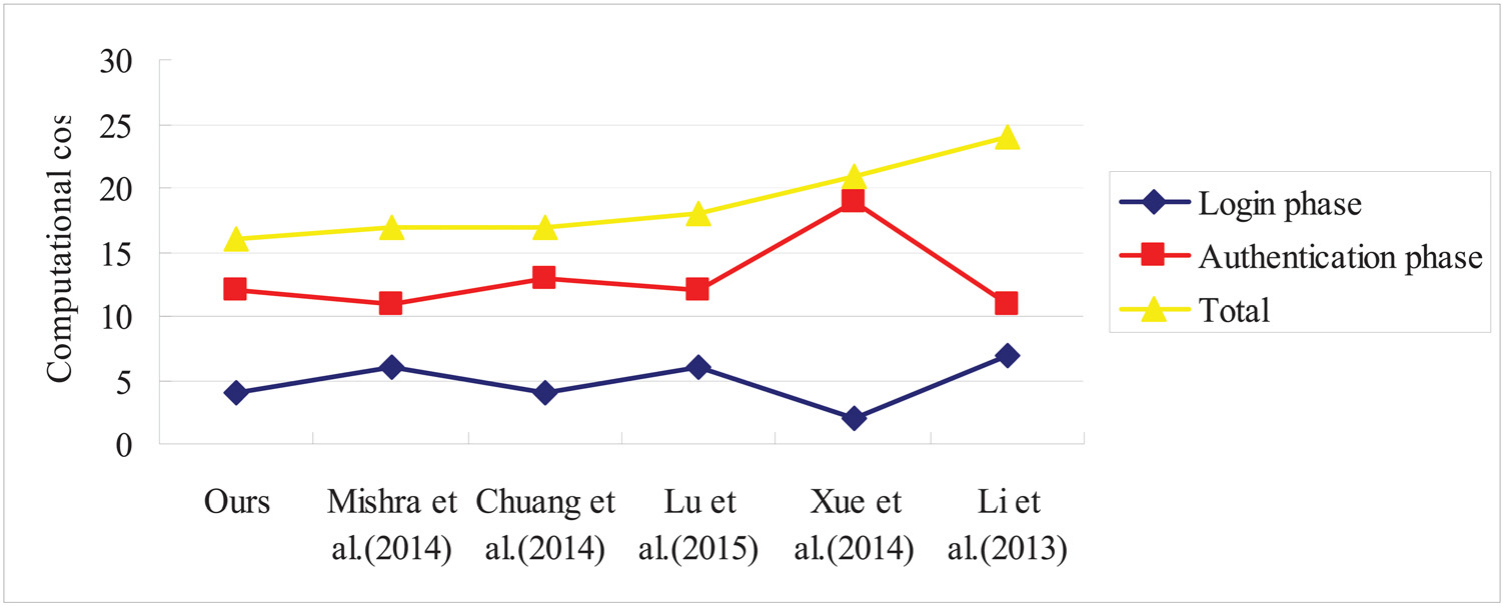
Performance comparison.

For performance analysis, we compare the computational primitives involved in login and authentication phases of our scheme and other related schemes. To analyze the computational complexity of the schemes, we use hashing operation as the time complexity since XOR operations require very little computations. Fig 2 shows comparison regarding the performance. From this comparison, we can see that our proposed scheme has better efficiency in comparison with other schemes.

## Conclusion and future work

In this paper, we presented a cryptanalysis of a recently proposed Mishra et al.’scheme and showed that their scheme was susceptible to replay attack while failed to provide an efficient password change phase. An improved scheme is proposed that inherits the merits of Mishra et al.’s scheme and resists different possible attacks. The proposed scheme is practical and efficient compared with other related schemes. Comprehensive security analysis proves that the robustness of our scheme is more secure than other related schemes. Among the open problems to be faced in the near future we can mention the study of specific applications and practical limitations of our scheme for mutual authentication using smart cards based on biometrics and their large-scale implementation in real multi-sever environments.
